# Diagnostic accuracy of contrast-enhanced computed tomography in assessing bone invasion in patients with oral squamous cell carcinoma

**DOI:** 10.1007/s00784-024-05705-3

**Published:** 2024-05-15

**Authors:** Ann-Kristin Struckmeier, Mayte Buchbender, Abbas Agaimy, Marco Kesting

**Affiliations:** 1 Department of Oral and Cranio-Maxillofacial Surgery, Friedrich-Alexander-Universität Erlangen- Nürnberg (FAU), Glückstraße 11, 91054 Erlangen, Germany; 2 Department of Oral and Maxillofacial Surgery, University Medical Center Hamburg-Eppendorf, Hamburg, Germany; 3 Institute of Pathology, Friedrich-Alexander-Universität Erlangen-Nürnberg (FAU), Erlangen, Germany

**Keywords:** Computed tomography, Bone invasion, Diagnostic accuracy, Oral squamous cell carcinoma

## Abstract

**Objectives:**

This study aimed to evaluate the diagnostic accuracy of contrast-enhanced computed tomography (CT) in detecting bone invasion in oral squamous cell carcinoma (OSCC) patients and to explore clinicopathological factors associated with its reliability.

**Materials and methods:**

417 patients underwent preoperative contrast-enhanced CT followed by radical surgery. The presence or absence of bone invasion served as the outcome variable, with histopathologic examination of the resection specimen considered the gold standard. Statistical analyses, comprising correlation analyses and the determination of sensitivity, specificity, positive predictive value (PPV), and negative predictive value (NPV), were conducted.

**Results:**

CT exhibited 76.85% sensitivity, 82.20% specificity, 47.14% PPV, and 89.67% NPV. False-positive and false-negative rates were 11.27% and 5.99%, respectively. Artifacts affected assessment in 44 patients, but not in those with bone invasion. Tumor size, depth of invasion (DOI), tumor localization at the upper jaw, lymphatic invasion, and perineural invasion correlated with incorrect identification of bone invasion (Chi-square, *p* < 0.05).

**Conclusions:**

Despite utilizing thin-section CT, notable false-positive and false-negative results persisted. Patients with T3 tumors, DOI ≥ 10 mm, or upper jaw tumors are at higher risk for misidentification of bone invasion. Combining multiple methods may enhance diagnostic accuracy, and the integration of artificial intelligence or tracking electrolyte disturbances by tumor depth profiling shows promise for further assessment of bone invasion before histopathology.

**Clinical relevance:**

Surgeons should consider these insights when planning tumor resection. Supplementary imaging may be warranted in cases with high risk factors for misidentification. Further methodological advancements are crucial for enhancing diagnostic precision.

**Supplementary Information:**

The online version contains supplementary material available at 10.1007/s00784-024-05705-3.

## Introduction

The established gold standard for treating oral squamous cell carcinoma (OSCC) encompasses surgical resection with local wide excision, guaranteeing a sufficient safe margin, and concurrent neck dissection (ND). The required extent of resection is greatly influenced by the presence of bone invasion.

However, a significant challenge in intraoperative bone invasion detection stems from the limitation that, unlike diagnostic methods applicable to assessing resection margins in soft tissue or lymph node metastases (LNMs), the frozen section technique is not suitable for bone diagnostics. This is due to the requirement for bones to undergo a decalcification process spanning several days before they can undergo histological evaluation [1]. Consequently, effective treatment planning relies significantly on a thorough preoperative assessment of bone invasion.

The prevalence of bone involvement in OSCC patients varies, ranging from 12 to 56% [2–4]. Bone involvement primarily results from the direct infiltration by the tumor. The main route of entry is reported to be through the alveolar crest and lingual cortex when the tumor is located medially to the mandible [5, 6]. Other routes of infiltration are also described, including spread through the canal of the inferior alveolar nerve in the mandible. Direct bone involvement by OSCC occurs in two main patterns: erosive and infiltrative. Erosive involvement takes place when the cortical bone recedes before a pushing tumor border [7]. In this form of involvement, there is frequently a scalloped excavation of the underlying medullary bone. In the infiltrative pattern of tumor involvement, cancer diffusely spreads throughout the cancellous and medullary bone [7, 8].

Clinical examination is pivotal in the detection of bone invasion, with a significant indicator being the fixation of the tumor to the bone upon palpation. Furthermore, adhering to the German guideline for OSCC therapy, preoperative contrast-enhanced computed tomography (CT) or magnetic resonance imaging (MRI) should be conducted to determine the local extent of the tumor and identify potential bone invasion, as well as assess LNMs [9, 10]. However, a prevalent challenge in preoperative assessment is the occurrence of frequent beam-hardening artifacts, often induced by implants or metal dental fillings, which can compromise the clarity of the CT image. Thereby, visualizing bone invasion may be more straightforward in edentulous patients compared to dentate patients [11]. Nonetheless, a common sign of neoplastic invasion into the medullary cavity is the identification of cortical defects [12, 13].

As mentioned earlier, the preoperative evaluation of bone invasion holds substantial importance in deciding the required scope of bone resection for oncological safety in patients with OSCC. When bone invasion is identified, indicating infiltration into the bone, a segmental mandibulectomy becomes a necessary procedure. Conversely, when the tumor is only attached to the lower jaw, indicative of erosive mandibular involvement, only a marginal mandibulectomy is deemed essential. Failing to identify superficial bone invasion may lead to insufficient marginal bone resection and the need for reoperation. On the contrary, segmental mandibulectomy requires a significant reconstructive procedure for both cosmetic and functional reasons [14, 15]. Thus, to enhance the quality of life, it is crucial to maintain the continuity of the mandible whenever it is oncologically safe to do so [16].

However, the existing body of knowledge presents a diverse range of data concerning the reliability of CT imaging in accurately identifying bone invasion in OSCC patients preoperatively. The main objective of this study was to evaluate the accuracy of contrast-enhanced CT in detecting bone invasion in OSCC patients by comparing preoperative CT imaging results with subsequent histopathological findings, especially the type of bone invasion. Additionally, we conducted comparative analyses to further explore the impact of various clinicopathological characteristics on the diagnostic accuracy of CT.

## Materials and methods

### Study design and participants

A retrospective study was conducted on a cohort of patients diagnosed with primary OSCC. All patients underwent staging with contrast-enhanced CT and received primary surgical treatment, which included tumor resection and ND as well as microvascular defect closure. Marginal resection was carried out in cases where the tumor caused bone erosion, while segmental resection was performed in cases of bone infiltration. Subsequently, histopathological examination of all tissue specimens took place at the Department of Oral and Maxillofacial Surgery at the University Hospital Erlangen.

The treatment protocol followed the national OSCC therapy guidelines. Diagnoses were made between January 1, 2013, to May 31, 2023. Patients with recurrent OSCC and those who did not undergo ND or had a reduced extent of ND due to severe comorbidities were excluded.

A comprehensive set of parameters was recorded and evaluated, including age, sex, tumor localization, clinical and pathological tumor, node, metastasis (TNM) classification, depth of invasion (DOI), histological grading, presence of perineural, lymphatic, or vascular invasion, and the type of bone invasion. All characteristics were extracted from hospital medical records. The TNM classification underwent revision during the study period. To maintain consistency in our findings [17], we reclassified patients initially categorized under the 7th TNM classification before 2017. As a result, all patients were categorized based on the 8th TNM classification.

The tissue samples were sent to the Department of Pathology for histopathological analysis. The TNM classification, all histopathological parameters, and the type of bone invasion were provided by the Department of Pathology at the University Hospital Erlangen.

Following national and institutional regulations, written informed consent was not deemed necessary from the participating patients. The study’s design and methods received approval from the Ethics Committee of Friedrich-Alexander University Erlangen-Nuremberg (Ethic votes: 23-185-Br, 23-186-Br).

Our study adhered to the Standards of Reporting of Diagnostic Accuracy (STARD) reporting guideline for diagnostic studies.

### Contrast-enhanced computed tomography

All patients included in this study underwent thin-section axial multidetector CT scans, employing a minimal slice thickness of 1 mm. Furthermore, sagittal and coronal multiplanar reconstructions with a slice thickness of 3 mm were generated using soft-tissue and bone algorithms. The CT scanners employed were SOMATOM Definition AS + and SOMATOM X.ceed from Siemens Healthineers (Erlangen, Germany).

Every CT scan was conducted with the administration of intravenous iodine-based contrast agent (Imeron 350 mg/mL, Bracco Group, Milan, Italy) to improve the differentiation of soft tissues, with a flow rate of 3 mL/s.

The evaluation of CT datasets involved a minimum of two independent physicians from the Department of Radiology. At least one consultant assessed the local extent of the tumor and evaluated bone invasion.

### Statistical analysis

Statistical analysis was conducted using the Statistical Package for the Social Sciences 28.0 (SPSS, Chicago, IL, USA).

Descriptive statistics were represented through frequency tables, crosstabs, and bar charts. Categorical variables were expressed as absolute and relative frequencies. Relationships between different characteristics were determined using cross tables, with the probabilities of correlations checked through the chi-square test.

The diagnostic accuracy of contrast-enhanced CT imaging was assessed by calculating sensitivity, specificity, positive predictive value (PPV), and negative predictive value (NPV), with histopathological results serving as the gold standard.

Generally, a *p* value < 0.05 was considered statistically significant.

Figures were generated using SPSS.

## Results

### Patient cohort

The study encompassed a cohort of 417 individuals with primary OSCC. Among these, 258 (61.87%) were male, and 159 (38.13%) were female. The mean age was 64.72, with a standard deviation of 12.05. Tumor localization was predominantly observed at the floor of the mouth (*n* = 147; 35.25%), tongue (*n* = 105; 25.18%), and lower jaw (*n* = 69; 16.55%).

The distribution of pathological tumor stages was as follows: 152 (36.45%) in T1, 106 (25.42%) in T2, 51 (12.23%) in T3, and 108 (25.90%) in T4a.

Histopathological examination revealed the absence of LNMs in 275 patients (65.95%), while 34.05% presented with metastatic disease.

Histopathological analysis unveiled that half of the patients had moderately differentiated carcinomas (51.32%, 214 patients), while 37.41% exhibited poorly differentiated carcinomas (156 patients), and only 9.59% displayed well-differentiated carcinomas (40 patients). Furthermore, histopathological analysis revealed lymphatic invasion in 8.15% (34 patients), vascular invasion in 2.40% (10 patients), and perineural invasion in 19.66% of the tumors (82 patients). Microscopically positive margins were observed in 1.92% of cases (8 patients).

The clinicopathological characteristics of the patient cohort are summarized in Table S1.

### Diagnostic accuracy of contrast-enhanced computed tomography

Next, we examined the diagnostic accuracy of contrast-enhanced CT regarding detection of bone invasion.

Overall, CT demonstrated a sensitivity of 76.85%, specificity of 82.20%, PPV of 47.14%, and NPV of 89.67%. CT accurately identified bone invasion in 83 and correctly ruled out bone invasion in 242 patients. Artifacts rendered CT imaging unassessable in 44 patients. Thereby, CT yielded 47 (11.27%) false-positive and 25 (5.99%) false-negative results.

The results are depicted in Table 1.

**Table 1:** Diagnostic accuracy of computed tomography in assessing bone invasion in patients with oral squamous cell carcinoma

Sensitivity	Specificity	Positive predictive value	Negative predictive value
76.85%	82.20%	63.85%	89.67%

### Correlation of clinicopathological characteristics with correctly or wrong identified bone invasion

Subsequently, a correlation analysis was undertaken to examine the relationship between incorrectly and correctly identified instances of bone invasion and the clinicopathological characteristics of the patients. Precise identification of bone invasion was associated with lower tumor stages, with the most challenging identification observed in tumors classified as pT3 (*p* < 0.001, false positive in patients with T3 tumors: 23 (45.10%), overall: 47 (36.15%)).

However, only in the groups of patients with pT1 and pT2 tumors a significant percentage of tumors could not be displayed in CT because of artifacts (19.08% and 14.15%).

Additionally, tumor localization at the upper jaw was associated with the incorrect identification of bone invasion (*p* = 0.032). Additionally, the misidentification of bone invasion demonstrated correlations with both lymphatic invasion (*p* = 0.010) and perineural invasion of the tumor (*p* < 0.001).

As the DOI increased, there was a notable decrease in the percentage of tumors correctly identified as either having or lacking bone invasion (*p* < 0.001).

**Fig. 1:**
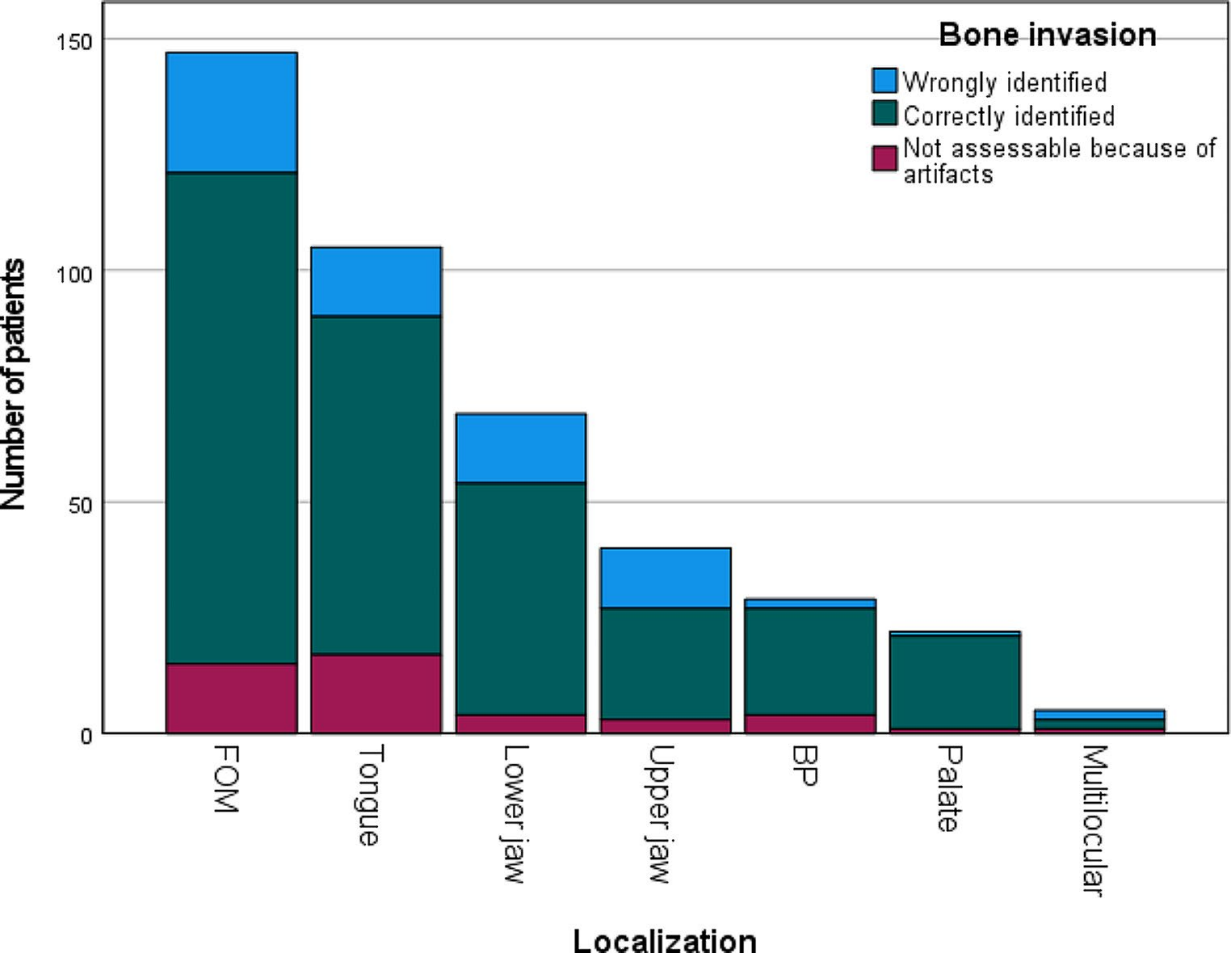
Number of patients with wrongly and correctly identified bone invasion depending on tumor localization Abbreviations. FOM = floor of the mouth, BP = buccal plane

**Fig. 2:**
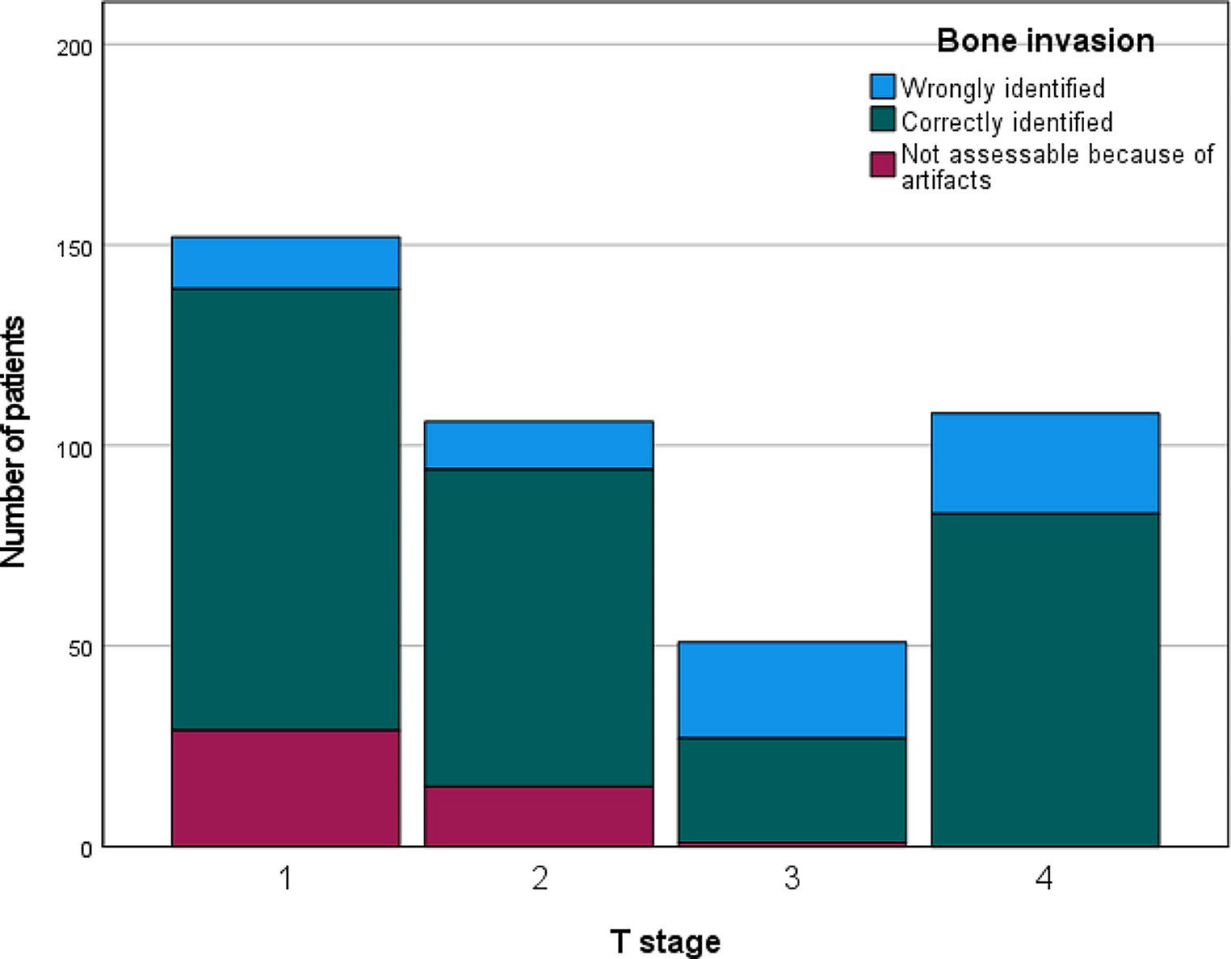
Number of patients with wrongly and correctly identified bone invasion depending on T stage

**Fig. 3:**
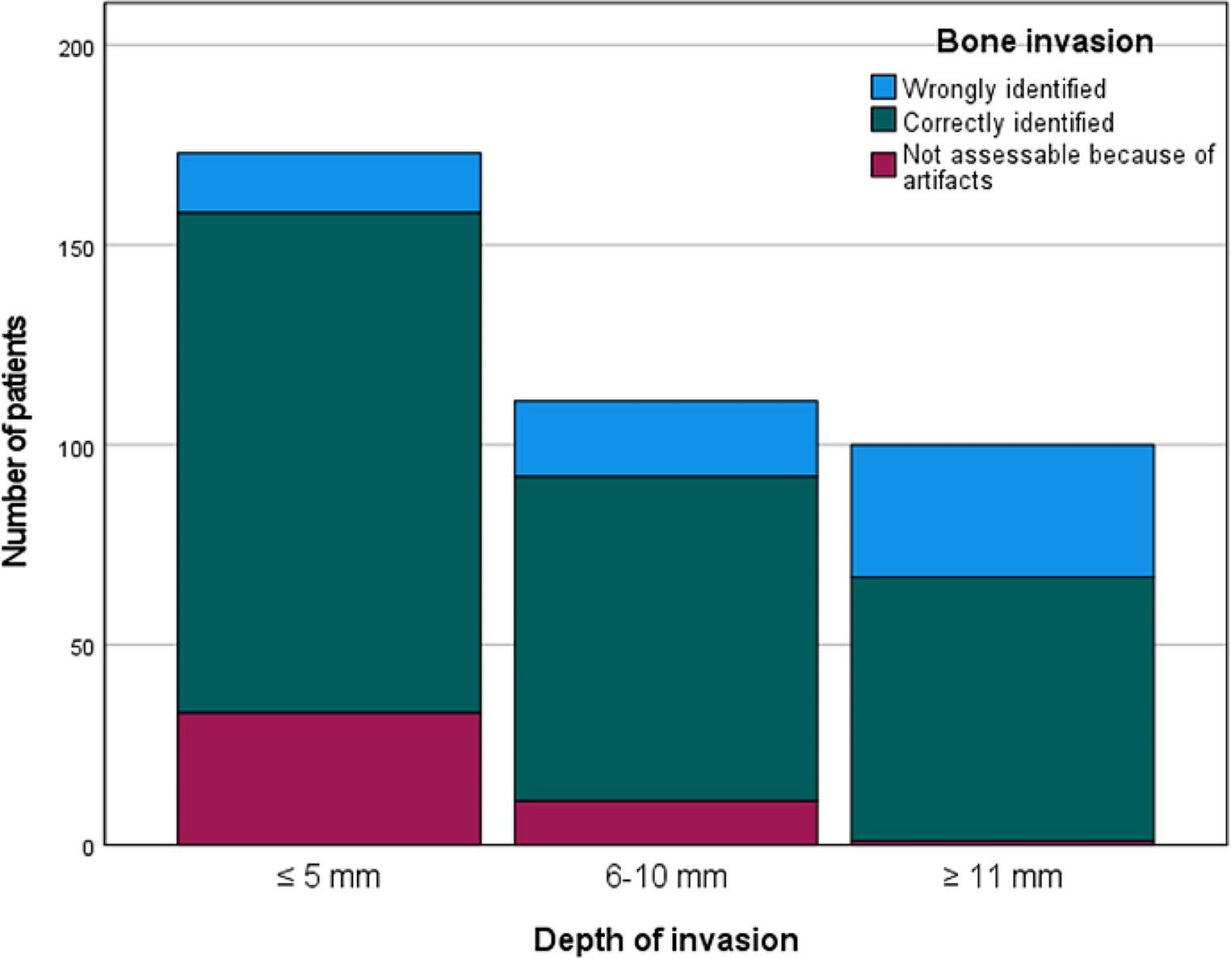
Number of patients with wrongly and correctly identified bone invasion depending on depth of invasion

Please see Tables 2 and 3 for the results of the statistical analysis. Moreover, results are graphically presented in Figs. 1, 2 and 3.

**Table 2:** Concordance of computed tomography with histopathological assessment regarding bone invasion depending on clinicopathological characteristics

Clinicopathological characteristics		Wrongly identified bone invasion (%)	Correctly identified bone invasion (%)	Correlation (Chi-square)
**Sex**	**Male**	45 (19.48)	186 (80.52)	0.799
	**Female**	29 (20.57)	112 (79.43)	
**Age**	**< 65 years**	21 (21.43)	77 (78.57)	0.439
	**≥ 65 years**	18 (17.14)	87 (82.86)	
**Pathological tumor stage**	**T1**	13 (10.57)	110 (89.43)	< 0.001*
	**T2**	12 (13.19)	79 (86.81)	
	**T3**	24 (48.00)	26 (52.00)	
	**T4a**	25 (23.15)	83 (76.85)	
**Localization**	**Floor of the mouth**	26 (19.70)	106 (80.30)	0.032*
	**Tongue**	15 (17.05)	73 (82.95)	
	**Lower jaw**	15 (23.08)	50 (76.92)	
	**Upper jaw**	13 (35.14)	24 (64.86)	
	**Buccal plane**	2 (8.00)	23 (92.00)	
	**Palate**	1 (4.76)	20 (95.24)	
	**Multilocular**	2 (50.00)	2 (50.00)	
**Grading**	**G1**	5 (16.13)	26 (83.87)	0.510
	**G2**	35 (18.13)	158 (81.87)	
	**G3**	32 (22.70)	109 (77.30)	
**Lymphovascular invasion**	**L0**	62 (18.34)	276 (81.66)	0.010*
	**L1**	12 (37.50)	20 (62.50)	
**Vascular invasion**	**V0**	71 (19.72)	289 (80.28)	0.423
	**V1**	3 (30.00)	7 (70.00)	
**Perineural invasion**	**Pn0**	48 (16.38)	245 (83.62)	< 0.001*
	**Pn1**	26 (33.77)	51 (66.23)	
**Depth of invasion**	**≤ 5 mm**	15 (10.71)	125 (89.29)	< 0.001*
	**6–10 mm**	19 (19,00)	81 (81.00)	
	**≥ 11 mm**	33 (33.33)	66 (66.67)	

**Table 3:** Bone invasion according to preoperative staging with computed tomography and pathological tumor stages

	Bone invasion according to computed tomography		
Pathological T stage	No (%)	Yes (%)	Artifacts (%)
**T1**	111 (73.03)	12 (7.89)	29 (19.08)
**T2**	79 (74.53)	12 (11.32)	14 (14.15)
**T3**	27 (52.94)	23 (45.10)	1 (1.96)
**T4a**	25 (23.15)	83 (76.85)	0 (0.00)

### Correlation of the type of bone invasion with the computed tomography results

**Fig. 4:**
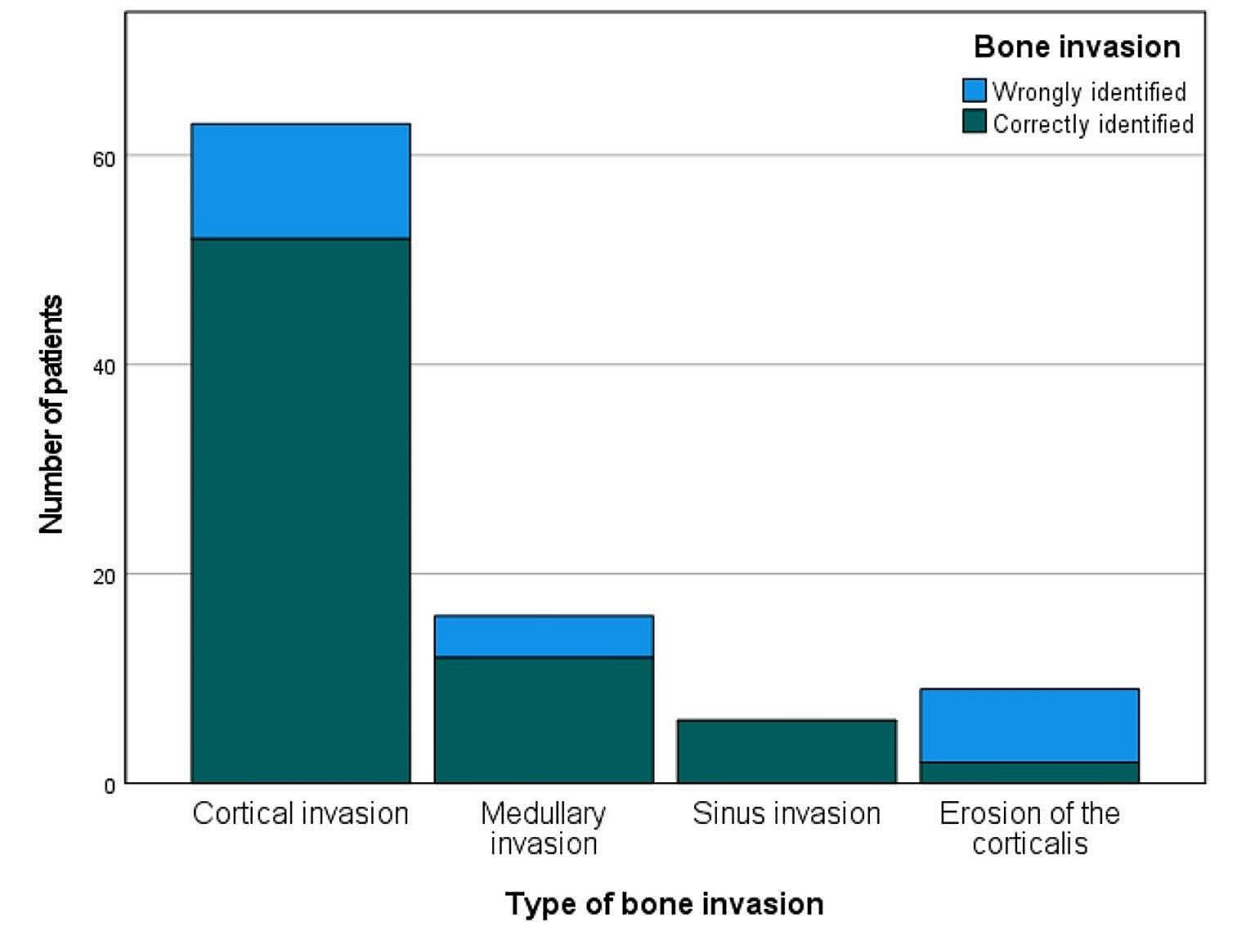
Number of patients with wrongly and correctly identified bone invasion depending on type of bone invasion

Histopathological analysis unveiled cortical invasion and medullary invasion in 63 and 16 tumors. Interestingly, a higher percentage of tumors with a cortical invasion were correctly identified as either having or lacking bone invasion than tumors with a medullary bone invasion (82.54% vs. 75.00%). Not surprisingly, bone invasion of tumors with sinusoidal invasion was correctly identified in 100% (6 tumors). Results are displayed in Table 4; Fig. 4.

**Table 4:** Diagnostic accuracy of computed tomography regarding bone invasion depending on type of bone invasion

Bone invasion	Wrongly identified bone invasion (%)	Correctly identified bone invasion (%)	Correlation (Chi-square)
**Cortical invasion**	11 (17.46)	52 (82.54)	0.002*
**Medullary invasion**	4 (25.00)	12 (75.00)	
**Invasion to the sinus**	0 (0.00)	6 (100.00)	
**Arrosion of the bone**	7 (77.78)	2 (22.22)	

## Discussion

Precise preoperative assessment of bone invasion is crucial for determining the extent of bone resection required in patients diagnosed with OSCC. Therefore, this study aimed to assess the diagnostic accuracy of contrast-enhanced CT in detecting bone invasion and to explore clinicopathological factors, particularly the type of bone invasion, associated with its reliability.

In our study, CT demonstrated a sensitivity of 76.85%, specificity of 82.20%, PPV of 47.14%, and NPV of 89.67%. CT accurately identified bone invasion in 83 and correctly ruled out bone invasion in 242 patients. Artifacts rendered CT imaging unassessable in 44 patients, but in none of the patients with bone invasion artifacts affected assessment. Thereby, CT yielded 47 (11.27%) false-positive and 25 (5.99%) false-negative results. Overall, the consistency of CT in detecting mandibular invasion varies. Czerwinka found a sensitivity and specificity of 86% and 68% [18]. Bouhir et al. found a sensitivity, specificity, PPV, and NPV of respectively 70%, 71%, 66%, and 76% for CT [19]. Wang et al. found a sensitivity and specificity in their meta-analysis of about 83% and 97%.

However, variations in results may arise due to distinct CT techniques. It is important to highlight that a significant portion of the existing data is over a decade old [20–22], thereby reducing its validity these days. This is attributed to the considerable advancements in the spatial resolution of CT scans, which have substantially enhanced the ability to detect small alterations. For example, Curran et al. utilized 4- to 5-mm-thick sections without routine assessment through bone algorithms and found a sensitivity of 89% as well as a specificity of 57% [23]. Similarly, Lane et al. employed 5-mm-thick sections without reconstructing with bone algorithms for assessment and found a sensitivity of 50% with a NPV of 61.1% and PPV of 91.1% [24]. Newer reports for CT reported a sensitivity and specificity of 69% and 80% [25] as well as 77% and 84% [26].

However, all patients included in this study underwent thin-section axial multidetector CT scans, employing a minimal slice thickness of 1 mm. Furthermore, sagittal and coronal multiplanar reconstructions with a slice thickness of 3 mm were generated and soft-tissue and bone algorithms were used.

However, high false-positive rates in our study might be attributed to periodontal disease [27]. The high false-negative rates may be related to studies suggesting that 50–75% of bone thickness must be missing for a cancellous defect to be detected [13].

The literature presents conflicting views on the superiority of CT or MRI for diagnosing primary oral cavity tumors. Some favor MRI for its higher sensitivity, while others assert CT’s superiority or equivalence. Slieker et al. compared CT and MRI in detecting maxillary bone invasion, finding CT more accurate (92% sensitivity, 87% specificity) than MRI (89% sensitivity, 58% specificity). However, the difference lacked statistical significance [28]. Chung et al. reported 100% sensitivity, 71% specificity, 50% PPV, and 100% NPV using MRI [29]. Bouhir found complementary roles for CT and MRI in mandibular bone assessment, with sensitivity, specificity and PPV and NPV of respectively, 83%, 50%, 59%, and 78% for MRI, and 83%, 62% 62%, 83% for associated CT and MRI [19]. Thereby, they concluded that CT and MRI are complementary for preoperative assessment of mandibular bone invasion, be it cortical and/or medullary, and in some cases may allow mandibular bone-sparing [19].

Patients generally prefer CT examinations due to their shorter duration and better tolerance compared to MRI [30]. Conversely, MRI excels in providing superior soft tissue contrast, detailed recognition of soft tissues and superficial structures, and is particularly advantageous in minimizing artifacts from metallic dental fillings or implants [31]. This improvement is evident in detecting perineural, intramuscular [30], or perivascular tumor extent, as well as assessing involvement of the skull base, orbit, or cervical spine. CT is considered preferable for evaluating cortical erosion [32], while MRI is employed for assessing bone marrow infiltration [33]. This observation was confirmed in our study with a higher percentage of tumors with a cortical invasion being correctly identified as either having or lacking bone invasion than tumors with a medullary bone invasion. However, in salvage surgery with previous external radiation, it should be noted that mucosal edema in MRI might reduce tumor tissue discrimination [34].

With advancements in medical technology, alternative diagnostic methods should be considered. Curran et al. explored single-photon emission computed tomography (SPECT) for bone invasion, reporting 100% sensitivity, 29% specificity, 64% PPV, and 100% NPV [23].

A systematic review compared several modalities in detecting mandibular invasion by OSCC, and the results showed that the sensitivity of bone invasion diagnosis for MRI, cone beam CT (CBCT), spiral CT, and panoramic radiography was 94%, 91%, 83%, and 55%, respectively, whereas the specificity was 100%, 100%, 97%, and 91.7%, respectively [27]. Brown et al. conducted a meta-analysis regarding accuracy of different methods and found sensitivities of 94% for bone scintigraphy, 93% for SPECT, 78% for MRI, and 72% for CT [35]. They proposed an initial staging with MRI and orthopantomogram, advising a CT scan if invasion is suggested and SPECT in cases of early invasion or uncertainty [35]. On the contrary, Buller et al. only found only small benefits using SPECT [26].

Furthermore, employing better-validated high-risk criteria may aid in determining which patients truly require additional imaging methods. In our study population, precise identification of bone invasion was notably associated with lower tumor stages, whereas it was particularly challenging in pT3 tumors (*p* < 0.001). The upper jaw tumor localization was linked to incorrect identification of bone invasion (*p* = 0.032). Additionally, misidentification of bone invasion correlated with vascular invasion (*p* = 0.010), and perineural invasion of the tumor (*p* < 0.001).

Moreover, a significant correlation was found with the DOI after histopathological assessment (*p* < 0.001). In line with our findings, Michcik et al. reported concordant results for T1 tumors in 62.5% of cases, T2 in 56.25%, T3 in 25%, and T4 in 42.9%. In their study cohort, concordance was 62% for cases with a DOI ≤ 10 mm and 33.3% for DOI > 10 mm. This underscores the growing difficulty of accurately delineating tumor boundaries, especially in large tumors exhibiting endophytic growth.

With advancements in medical technology, alternative methods should be considered. Enhancing the diagnostic accuracy of CT in predicting bone invasion among oncologic patients could be achieved through the utilization of artificial intelligence and deep learning models. In this context, research has already been conducted on staging bone malignancies [46]. Moreover, the validation of medical imaging tools is a topic of significant clinical interest. Achieving highly accurate coregistration between histopathological and radiological images, specifically concerning tumor boundaries, can offer enhanced clarity. A prospective study employing a well-defined diagnostic algorithm that delineates cortical and/or medullary invasion and periosteal reaction could provide valuable insights. In existing literature, individually analyzing these signs has shown diagnostic value. Notably, perimandibular periosteal reaction or cortical erosion on CT has been reported to be significantly associated with bone invasion on histology [36, 37].

Additionally, the detection of microscopic tumor spread in bone using tumor depth profiling could be based on tracking electrolyte disturbances, as they are critical contributors to tumor invasion in bone [38, 39].

### Limitations of the study

Our study presents several limitations that warrant consideration when interpreting the findings. Firstly, its retrospective and single-center design introduces inherent biases. However, it is crucial to recognize that past studies investigating the diagnostic accuracy of CT in assessing bone invasion have frequently encountered challenges stemming from smaller sample sizes or heterogeneous data. In contrast, our study stands out due to its significant sample size of 417 patients and a highly homogeneous patient cohort, which excludes other types of head and neck squamous cell carcinoma, thus setting it apart from similar investigations. Furthermore, unlike prior research, we specifically examined the diagnostic accuracy based on the type of bone invasion. Nonetheless, it is essential to interpret the results cautiously, given that the dentate condition may significantly influence outcomes and contribute to false-positive results. Interpretation of bone invasion might be more straightforward in edentulous patients than in dentate patients [11]. For instance, periodontitis could lead to false-positive results [5, 11].

## Conclusion

Despite utilizing thin-section CT with a minimal slice thickness of 1 mm and reconstruction with a bone algorithm, there remains a notable incidence of false-positive and false-negative results. Patients with T3 tumors, a DOI ≥ 10 mm, or tumors localized at the upper jaw are at high risk for wrong identification of bone invasion. Combining multiple methods may enhance diagnostic accuracy, and the integration of artificial intelligence or tracking electrolyte disturbances by tumor depth profiling shows promise for further assessment of bone invasion before histopathology. However, these prospects require additional investigation.

### Electronic supplementary material

Below is the link to the electronic supplementary material.

Supplementary Material 1

## Data Availability

The data that support the findings of this study are available from the corresponding author upon reasonable request.

## Abbreviations

CT: computed tomography
DOI: depth of invasion
OSCC: oral squamous cell carcinoma
LNM: lymph node metastasis
MRI: magnetic resonance imaging
ND: neck dissection
NPV: negative predictive value
PPV: positive predictive value
TNM: tumor, node, metastasis
