# Supplementary material for: Diagnostic accuracy of contrast-enhanced computed tomography in assessing bone invasion in patients with oral squamous cell carcinoma
Source: Clin Oral Investig. 2024 May 15;28(6):314. doi: 10.1007/s00784-024-05705-3 (PMC11096202; doi:10.1007/s00784-024-05705-3)
Supplement: Supplementary file 1 — Supplementary Material 1 [file 784_2024_5705_MOESM1_ESM.docx]

**Supplemental material:** **Diagnostic accuracy of contrast-enhanced computed tomography in assessing bone invasion in patients with oral squamous cell carcinoma**

Ann-Kristin Struckmeier MD, DMD, Mayte Buchbender DMD, Abbas Agaimy MD, Marco Kesting MD, DMD

**Table S1.** Clinicopathological characteristics of the investigated cohort

| **Characteristics** | **Number of patients (%)** |
| --- | --- |
| **No. of patients** | 417 |
| **Sex** | |
| Male | 258 (61.87) |
| Female | 159 (38.13) |
| **Age** | |
| Mean | 64.72 |
| Standard Deviation | 12.05 |
| **Tumor localization** | |
| Floor of the mouth | 147 (35.25) |
| Tongue | 105 (25.18) |
| Lower jaw | 69 (16.55) |
| Upper jaw | 40 (9.59) |
| Buccal plane | 29 (6.95) |
| Palate | 22 (5.28) |
| Multilocular | 5 (1.20) |
| **Pathological tumor stage** | |
| T1 | 152 (36.45) |
| T2 | 106 (25.42) |
| T3 | 51 (12.23) |
| T4a | 108 (25.90) |
| **Pathological nodal stage** | |
| N0 | 275 (65.95) |
| N1 | 44 (10.55) |
| N2a | 11 (2.64) |
| N2b | 28 (6.71) |
| N2c | 11 (2.64) |
| N3b | 48 (11.51) |
| **Histological grading** | |
| G1 | 40 (9.59) |
| G2 | 214 (51.32) |
| G3 | 156 (37.41) |
| Gx | 7 (1.68) |
| **Lymphatic invasion** | |
| L0 | 381 (91.37) |
| L1 | 34 (8.15) |
| Lx | 2 (0.48) |
| **Vascular invasion** | |
| V0 | 405 (97.12) |
| V1 | 10 (2.40) |
| Vx | 2 (0.48) |
| **Perineural invasion** | |
| Pn0 | 333 (79.86) |
| Pn1 | 82 (19.66) |
| Pnx | 2 (0.48) |
| **Residual tumor** | |
| R0 | 406 (97.36) |
| R1 | 8 (1.92) |
| Rx | 3 (0.72) |
| **Depth of invasion** |  |
| ≤ 5 mm | 173 (41.49) |
| 6-10 mm | 111 (26.62) |
| **≥** 10 mm | 100 (23.98) |
| DOIx | 33 (7.91) |
